# Comparative predictive values of anthropometric indices for cardiometabolic multimorbidity in middle-aged and older adults: a prospective study from the CHARLS study

**DOI:** 10.1186/s12889-025-26060-2

**Published:** 2025-12-23

**Authors:** Leiming Zhang, Zi Liu, Dan Si, Haitao Yang, Xianwei Fan, Lijie Yan, Jingjing Liu, Xuejie Li, Juan Hu, Jintao Wu

**Affiliations:** 1 Fuwai Central China Cardiovascular Hospital, Zhengzhou, China; 2 Henan Provincial People’s Hospital, People’s Hospital of Zhengzhou University, Zhengzhou, China

**Keywords:** Cardiometabolic multimorbidity, Anthropometric indices, Body mass index, Waist circumference

## Abstract

**Objectives:**

This study aimed to compare the predictive performance of seven anthropometric indices—body mass index (BMI), waist-to-height ratio (WHtR), body roundness index (BRI), weight-adjusted waist index (WWI), a body shape index (ABSI), conicity index (CI) and waist circumference (WC)—for cardiometabolic multimorbidity (CMM) in middle-aged and older Chinese adults.

**Methods:**

This study conducted a prospective study using data from the China Health and Retirement Longitudinal Study (CHARLS) 2011–2018. Propensity score matching (PSM) was utilized to control for biases induced by age and gender, with these two factors as the core matching variables and sample matching conducted at a 1:1 ratio. Multivariable logistic regression models were used to examine associations between anthropometric indices and CMM. Restricted cubic splines explored dose-response relationships between anthropometric indices and CMM. Receiver operating characteristic (ROC) curves evaluated discriminative performance of anthropometric indices in predicting CMM and specific types of CMM.

**Results:**

Before PSM, a total of 7,469 participants were included, 554 participants (7.42%) developed CMM. In Model II, BMI, WHtR, BRI, CI and WC maintained significant associations across higher quartiles. Compared with the BMI *Q*1 group, the risk of CMM in *Q*2 group increased by 1.55 times (OR = 2.55, 95%CI = 1.65, 3.93, *P* < 0.001); the risk in *Q*3 group increased by 2.04 times (OR = 3.04, 95%CI = 1.93, 4.81, *P* < 0.001); and the risk in *Q*4 group increased by 4.89 times (OR = 5.89, 95%CI = 3.62, 9.57, *P* < 0.001). Compared with the WHR *Q*1 group, the risk of CMM in *Q*2 group increased by 1.26 times (OR = 2.26, 95%CI = 1.49, 3.42, *P* < 0.001); the risk in *Q*3 group increased by 1.54 times (OR = 2.54, 95%CI = 1.65, 3.91, *P* < 0.001); and the risk in *Q*4 group increased by 3.54 times (OR = 4.54, 95%CI = 2.87, 7.16, *P* < 0.001). Similar results were found in BRI. Compared with the CI *Q*1 group, the risk in *Q*3 group increased by 0.59 times (OR = 1.59, 95%CI = 1.04, 2.44, *P* = 0.032); and the risk in *Q*4 group increased by 0.73 times (OR = 1.73, 95%CI = 1.11, 2.70, *P* = 0.015). Compared with the WC *Q*1 group, the risk of CMM in *Q*2 group increased by 0.82 times (OR = 1.82, 95%CI = 1.19, 2.80, *P* = 0.006); the risk in *Q*3 group increased by 1.36 times (OR = 2.36, 95%CI = 1.51, 3.68, *P* < 0.001); and the risk in *Q*4 group increased by 4.63 times (OR = 5.63, 95%CI = 3.46, 9.15, *P* < 0.001). WHtR, BRI, WWI, CI and WC all showed a U-shaped association with CMM risk. BMI demonstrated a linear relationship with CMM risk. BMI achieved the highest performance with identical AUC values of 0.720 (0.690–0.749), followed by WC with an AUC of 0.712 (0.682–0.742). BMI and WC exhibited superior predictive performance whether in predicting those specific types of CMM.

**Conclusion:**

BMI and WC were superior to novel anthropometric indices for CMM risk prediction in middle-aged and older Chinese adults. The finding supports their value in identifying high-risk CMM individuals and reinforces their role as practical tools.

## Introduction

 Cardiometabolic multimorbidity (CMM), defined as the coexistence of two or more conditions from diabetes, heart disease, and stroke, represents a major public health challenge worldwide [1]. CMM increases the risk of various adverse health outcomes, especially in middle-aged and older populations [2, 3]. The study found that the prevalence of CMM was 14.4% among the North American population, 7.45% among the Nordic population, and 5.02% among the East Asian population [4]. In the Chinese population, the prevalence of CMM was 3.07% [5]. Another study found that patients with CMM had a 1.21-fold higher all-cause mortality rate than those without CMM [6]. CMM not only affects patient rehabilitation but also has an adverse impact on patient prognosis [2]. Therefore, identifying those at high risk of developing CMM at an early stage is critical for carrying out delivering targeted prevention strategies.

Anthropometric indices have emerged as potentially valuable screening tools for cardiometabolic risk assessment due to their simplicity, non-invasiveness, and cost-effectiveness [7]. The body mass index (BMI) remains the most commonly employed indicator of adiposity [8–10]. Waist circumference (WC) is a commonly used indicator for assessing body fat distribution, primarily reflecting the accumulation of abdominal fat (i.e., central obesity) through its measurement [11]. Besides, several alternative anthropometric indices have been developed to capture body fat distribution-especially central adiposity, which is associated with unfavorable cardiometabolic results [12]. Waist-to-height ratio (WHtR) has demonstrated superior performance to BMI in predicting hypertension in Chinese adults [13]. Body roundness index (BRI), a relatively new index introduced by Thomas et al., incorporates both WC and height to estimate whole-body fat percentage and visceral adipose tissue [14]. Weight-adjusted waist index (WWI), proposed by Park Y. et al., adjusts WC for the effect of body weight, potentially providing a more refined measure of central adiposity [15]. A body shape index (ABSI), developed by Krakauer and Krakauer, combines WC, weight, and height to identify excess abdominal fat independently of BMI [16]. Conicity index (CI) uses these same parameters (WC, weight, and height) to assess abdominal fat distribution based on the geometric concept of a cylinder to cone transition [17]. Previous studies in Chinese populations have examined the relationship between various anthropometric indices and individual cardiometabolic diseases. For instance, Chinese scholars found that a rise in WWI was significantly linked to an elevated occurrence of newly diagnosed type 2 diabetes among rural Chinese adults of Northeast China [18]. Similarly, in a large cross-sectional study of 44,048 Chinese adults, Liu et al. evaluated multiple anthropometric measurements and found that waist-related indices, particularly WHtR, had stronger predictive values for cardiometabolic risk factors and cardiovascular diseases compared to BMI [19]. However, studies comparing the predictive performance of these seven indices for CMM in Chinese middle-aged and older populations remain extremely scarce, many studies are limited to 3–4 indices. To fill this key research gap, this study utilizes longitudinal data from the China Health and Retirement Longitudinal Study (CHARLS, 2011–2018) to systematically evaluate the predictive value of seven anthropometric indices (BMI, WC, WHtR, BRI, ABSI, WWI, and CI) for CMM among middle-aged and older Chinese adults.

## Methods

### Study subjects

The present study drew on data from CHARLS, a large-scale longitudinal survey focused on Chinese individuals aged 45 and above. The project collects comprehensive information on respondents’ health status, lifestyle, chronic diseases, income, wealth, and social security, providing valuable data for in-depth research on health and economic issues among middle-aged and elderly populations. As this study used a public database, it was exempt from ethical approval.

**Fig. 1:**
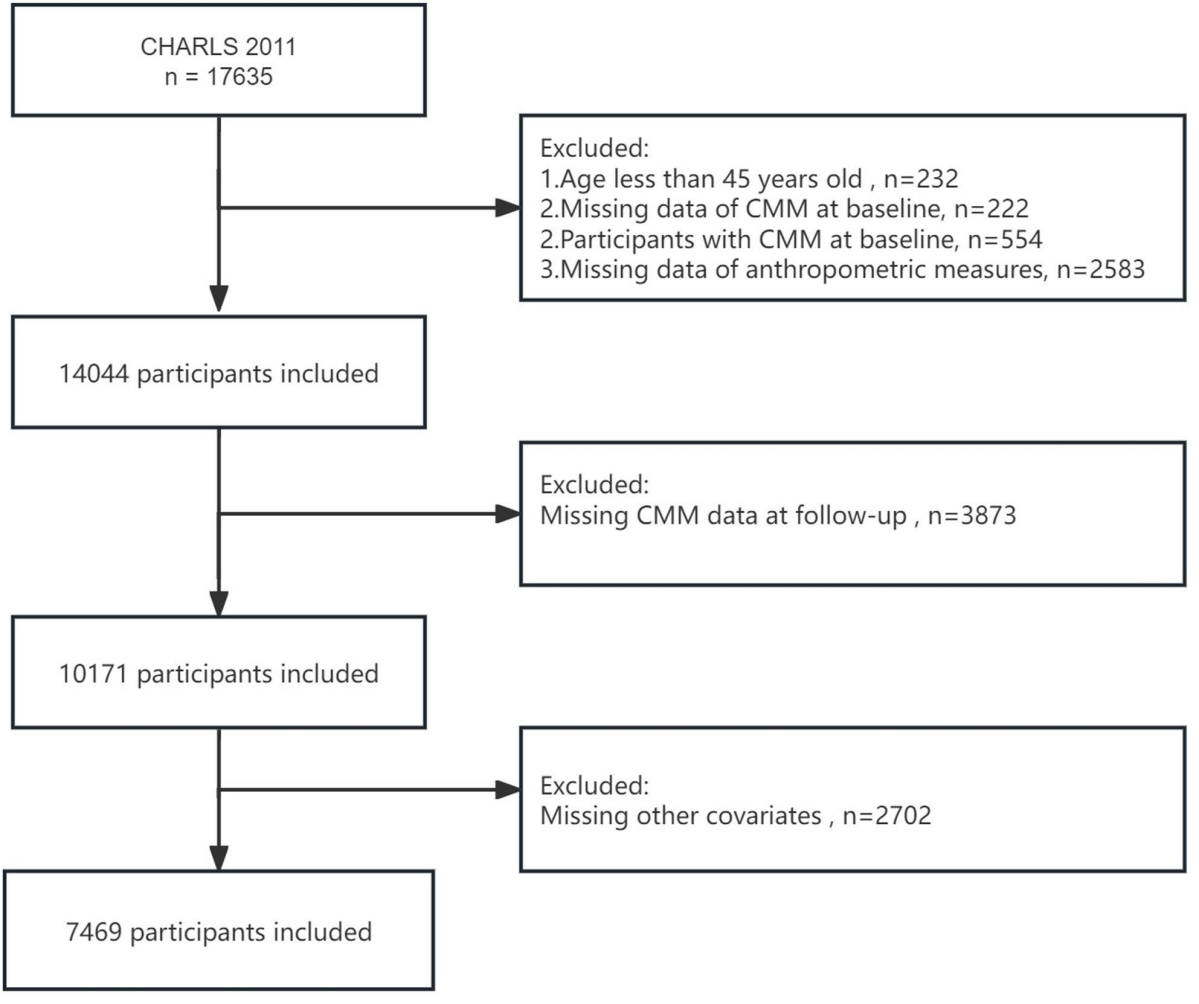
The flow chart of participants inclusion and exclusion in this study

17,635 participants were initially encompassed in the baseline survey. Participants were excluded based on the following criteria: (1) age < 45 years old (*N* = 232); (2) missing baseline CMM data (*N* = 222); (3) diagnosed with CMM at baseline (*N* = 554); (4) with incomplete anthropometric measurements (*N* = 2,583); (5) missing CMM data at follow-up (*N* = 3,873); (6) missing data on other covariates (*N* = 2,702). Ultimately, 7,469 participants were encompassed in the study (Fig. 1). The study design and procedures were conducted in accordance with the Declaration of Helsinki.

### Measurement of anthropometric indices

Body weight, height, and WC were measured by trained technicians during the baseline survey. Six anthropometric indices were calculated to reflect general and central adiposity: BMI, WHtR, BRI, WWI, ABSI, and CI. All indices were derived using the following formulas:

$$\:\mathrm{B}\mathrm{M}\mathrm{I}=\frac{\mathrm{w}\mathrm{e}\mathrm{i}\mathrm{g}\mathrm{h}\mathrm{t}}{{\mathrm{h}\mathrm{e}\mathrm{i}\mathrm{g}\mathrm{h}\mathrm{t}}^{2}}$$

$$\:\mathrm{W}\mathrm{H}\mathrm{t}\mathrm{R}=\frac{\mathrm{W}\mathrm{C}}{\mathrm{h}\mathrm{e}\mathrm{i}\mathrm{g}\mathrm{h}\mathrm{t}}$$

$$\:\mathrm{B}\mathrm{R}\mathrm{I}=364.2-365.5\times\:\sqrt{1-{\left(\frac{\frac{\mathrm{W}\mathrm{C}}{2\pi\:}}{0.5\times\:\mathrm{h}\mathrm{e}\mathrm{i}\mathrm{g}\mathrm{h}\mathrm{t}}\right)}^{2}}$$

$$\:\mathrm{W}\mathrm{W}\mathrm{I}=\frac{\mathrm{W}\mathrm{C}}{\sqrt{\mathrm{w}\mathrm{e}\mathrm{i}\mathrm{g}\mathrm{h}\mathrm{t}}}$$

$$\:\mathrm{A}\mathrm{B}\mathrm{S}\mathrm{I}=\frac{\mathrm{W}\mathrm{C}}{{\mathrm{B}\mathrm{M}\mathrm{I}}^{2/3}\times\:{\mathrm{h}\mathrm{e}\mathrm{i}\mathrm{g}\mathrm{h}\mathrm{t}}^{1/2}}$$

$$\:\mathrm{C}\mathrm{I}=\frac{\mathrm{W}\mathrm{C}}{0.109\times\:\sqrt{\frac{\mathrm{w}\mathrm{e}\mathrm{i}\mathrm{g}\mathrm{h}\mathrm{t}}{\mathrm{h}\mathrm{e}\mathrm{i}\mathrm{g}\mathrm{h}\mathrm{t}}}}$$

### Assessment of CMM events

The endpoint of this study was the occurrence of CMM events, and participants were divided into the CMM and non-CMM groups based on whether CMM events occurred. CMM was defined as the coexistence of at least two of the following cardiometabolic conditions: diabetes mellitus, heart disease, and stroke [20]. Heart disease and stroke were identified based on participants’ self-reported physician diagnoses in response to the following structured questions: “Have you ever been diagnosed with a heart problem (such as heart attack, angina, coronary heart disease, heart failure, or other heart conditions) by a doctor?” and “Have you ever been diagnosed with stroke by a doctor?” [21]. In addition to self-reported diabetes, participants were classified as having diabetes if any of the following criteria were met: fasting plasma glucose (FPG) ≥ 7.0 mmol/L; random blood glucose ≥ 11.1 mmol/L; hemoglobin A1c (HbA1c) ≥ 6.5% [22].

### Covariates

The covariates in this study included sociodemographic, health information and laboratory data. Sociodemographic variables included age, sex, marital status, and education level. Health information included diabetes, heart disease, stroke, hypertension, smoker and alcohol consumers. Laboratory test results included total cholesterol (TC), triglycerides (TG), high-density lipoprotein (HDL-C), low-density lipoprotein (LDL-C), estimated glomerular filtration rate (eGFR), serum uric acid (SUA), and HbA1c. Baseline data were collected in 2011 (Wave 1), and follow-up data were gathered in 2018 (Wave 4). Sociodemographic and health information were collected by questionnaires. Laboratory data were obtained by professional personnel after participants fasted for more than 12 h. It was not possible to determine the specific time of CMM onset in the patients included in this study.

### Statistical analysis

All analyses were performed using R software (version 4.3.0). To control for biases caused by age and gender, this study constructed a propensity score matching (PSM) model, with gender and age as the core matching variables, and completes sample matching at a 1:1 ratio. Continuous variables with normal distribution were presented as mean ± standard deviation (SD) and analyzed by independent samples t-test, while those with skewed distribution were described as median [interquartile range (IQR)] and tested by Wilcoxon rank-sum test. Categorical variables were expressed as numbers (percentages) and compared using the chi-square test. The associations between various anthropometric indices and CMM were assessed using multivariable logistic regression models. Results were reported as odds ratios (ORs) with corresponding 95% CIs. The crude model without adjustment; Model I, adjusted for age, sex, marital status, education level, smoking, alcohol consumption, diabetes, hypertension, heart disease, and stroke; and Model II, which was further adjusted for eGFR, HbA1c, TC, HDL-C, LDL-C, TG, and SUA. Restricted cubic spline (RCS) regression analyses were conducted to examine dose-response relationships between anthropometric indices and CMM, adjusting for all covariates included in Model II. The results were visualized with dose–response curves. To evaluate the differentiating ability of each anthropometric index in predicting CMM, receiver operating characteristic (ROC) curves were plotted, and the area under the curve (AUC), sensitivity, specificity, and Youden index were calculated. Furthermore, this study further explored the performance of anthropometric indices in predicting specific types of CMM. A two-sided *P* value < 0.05 was considered statistically significant.

## Results

### Baseline participant characteristics

Before PSM, 7,469 participants were encompassed, among whom 554 (7.42%) developed CMM during the follow-up period. The baseline characteristics of participants before PSM and after PSM were summarized in Table 1. After PSM, in terms of demographics, there were significant differences between the two groups in marital status and educational level (all *P* < 0.001), whereas there were no significant differences between the two groups in age and gender. As expected, the baseline prevalence of diabetes (29.24% vs. 8.12%, *P* < 0.001), heart disease (25.81% vs. 3.25%, *P* < 0.001), stroke (4.51% vs. 1.44%, *P =* 0.003), and hypertension (65.34% vs. 39.35%, *P* < 0.001) were all significantly higher in the CMM group after PSM. Regarding laboratory parameters, participants with CMM had higher levels of TC, LDL-C, TG, and HbA1c, but lower HDL-C values after PSM. In comparison with the non-CMM group, all anthropometric indices were notably higher in the CMM group except for ABSI after PSM. All subsequent analyses in this study were conducted based on the data after PSM.

**Table 1:** Baseline characteristics of the study participants before PSM and after PSM

	Before PSM				After PSM			
Characteristic	Total (*n* = 7469)	Non-CMM (*n* = 6915)	CMM (*n* = 554)	*P*-value	Total (*n* = 1108)	Non-CMM (*n* = 554)	CMM (*n* = 554)	*P*-value
Age, years	58.51 ± 8.77	58.371 ± 8.782	60.18 ± 8.43	< 0.001	60.18 ± 8.43	60.18 ± 8.43	60.18 ± 8.43	1.000
Sex, n(%)				0.001				1.000
Male	3360 (44.99%)	3147 (45.51%)	213 (38.45%)		426 (38.45%)	213 (38.45%)	213 (38.45%)	
Female	4109 (55.01%)	3768 (54.49%)	341 (61.55%)		682 (61.55%)	341 (61.55%)	341 (61.55%)	
Marital status, n(%)				0.837				< 0.001
Married	6666 (89.25%)	6173 (89.27%)	493 (88.99%)		968 (87.37%)	475 (85.74%)	493 (88.99%)	
Single/Divorced	803 (10.75%)	742 (10.73%)	61 (11.01%)		140 (12.63%)	79 (14.26%)	61 (11.01%)	
Education level, n(%)				0.539				< 0.001
High education	2243 (30.03%)	2083 (30.12%)	160 (28.88%)		238 (21.48%)	78 (14.08%)	160 (28.88%)	
Low education	5226 (69.97%)	4832 (69.88%)	394 (71.12%)		870 (78.52%)	476 (85.92%)	394 (71.12%)	
Smoking, n(%)								0.702
Yes	2823 (37.80%)	2636 (38.12%)	187 (33.76%)	0.041	368 (33.21%)	181 (32.67%)	187 (33.76%)	
No	4646 (62.20%)	4279 (61.88%)	367 (66.24%)		740 (66.79%)	373 (67.33%)	367 (66.24%)	
Alcohol consumers, n(%)				0.007				0.752
Yes	3037 (40.66%)	2842 (41.10%)	195 (35.20%)		385 (34.75%)	190 (34.30%)	195 (35.20%)	
No	4432 (59.34%)	4073 (58.90%)	359 (64.80%)		723 (65.25%)	364 (65.70%)	359 (64.80%)	
Diabetes, n(%)				< 0.001				< 0.001
Yes	875 (11.72%)	713 (10.31%)	162 (29.24%)		207 (18.68%)	45 (8.12%)	162 (29.24%)	
No	6594 (88.28%)	6202 (89.69%)	392 (70.76%)		901 (81.32%)	509 (91.88%)	392 (70.76%)	
Heart disease, n (%)				< 0.001				< 0.001
Yes	641 (8.58%)	498 (7.20%)	143 (25.81%)		161 (14.53%)	18 (3.25%)	143 (25.81%)	
No	6828 (91.42%)	6417 (92.80%)	411 (74.19%)		947 (85.47%)	536 (96.75%)	411 (74.19%)	
Stroke, n (%)				< 0.001				0.003
Yes	85 (1.14%)	60 (0.87%)	25 (4.51%)		33 (2.98%)	8 (1.44%)	25 (4.51%)	
No	7384 (98.86%)	6855 (99.13%)	529 (95.49%)		1075 (97.02%)	546 (98.56%)	529 (95.49%)	
Hypertension, n(%)				< 0.001				< 0.001
Yes	2865 (38.36%)	2503 (36.20%)	362 (65.34%)		580 (52.35%)	218 (39.35%)	362 (65.34%)	
No	4604 (61.64%)	4412 (63.80%)	192 (34.66%)		528 (47.65%)	336 (60.65%)	192 (34.66%)	
TC, mg/dL	193.64 ± 38.04	193.21 ± 37.94	199.0 ± 38.93	< 0.001	196.41 ± 38.25	193.82 ± 37.41	199.0 ± 38.93	0.024
HDL-C, mg/dL	51.42 ± 15.22	51.75 ± 15.25	47.22 ± 14.21	< 0.001	49.01 ± 14.53	50.79 ± 14.64	47.22 ± 14.21	< 0.001
LDL-C, mg/dL	116.82 ± 34.72	116.48 ± 34.43	121.01 ± 37.97	0.007	117.91 ± 36.91	114.81 ± 35.57	121.01 ± 37.97	0.005
TG, mg/dL	130.46 ± 94.13	128.56 ± 92.38	154.13 ± 111.15	< 0.001	140.33 ± 101.72	126.53 ± 89.32	154.13 ± 111.15	< 0.001
eGFR, ml/min/1.73㎡	96.52 ± 13.54	96.72 ± 13.46	93.99 ± 14.33	< 0.001	94.07 ± 13.97	94.15 ± 13.60	93.99 ± 14.33	0.856
HbA1c	5.24 ± 0.74	5.21 ± 0.68	5.63 ± 1.19	< 0.001	5.39 ± 1.02	5.14 ± 0.73	5.63 ± 1.19	< 0.001
SUA, mg/dL	4.40 ± 1.21	4.39 ± 1.21	4.48 ± 1.24	0.078	4.46 ± 1.20	4.44 ± 1.16	4.48 ± 1.24	0.525
BMI, Kg/㎡	23.58 ± 3.86	23.44 ± 3.79	25.36 ± 4.19	< 0.001	23.88 ± 4.06	22.41 ± 3.32	25.36 ± 4.19	< 0.001
WHtR	0.54 ± 0.08	0.53 ± 0.08	0.57 ± 0.09	< 0.001	0.55 ± 0.09	0.53 ± 0.08	0.57 ± 0.09	< 0.001
BRI	4.16 ± 1.52	4.11 ± 1.49	4.86 ± 1.68	< 0.001	4.41 ± 1.65	3.96 ± 1.50	4.86 ± 1.68	< 0.001
ABSI	0.08 ± 0.01	0.08 ± 0.01	0.08 ± 0.01	0.030	0.08 ± 0.01	0.08 ± 0.01	0.08 ± 0.01	0.511
WWI	11.04 ± 1.35	11.02 ± 1.34	11.29 ± 1.44	< 0.001	11.19 ± 1.42	11.09 ± 1.40	11.29 ± 1.44	0.020
CI	1.27 ± 0.15	1.27 ± 0.15	1.30 ± 0.16	< 0.001	1.28 ± 0.16	1.27 ± 0.15	1.30 ± 0.16	< 0.001
WC, cm	84.24 ± 12.55	83.83 ± 12.37	89.31 ± 13.62	< 0.001	85.33 ± 13.55	81.35 ± 12.27	89.31 ± 13.62	< 0.001

*PSM* Propensity score matching, *CMM* Cardiometabolic multimorbidity, *TC* Total cholesterol, *TG* Triglycerides, *HDL-C* High-density lipoprotein cholesterol, *LDL-C* Low-density lipoprotein cholesterol, *eGFR* Estimated glomerular filtration rate, *SUA* Serum uric acid, *HbA1* Hemoglobin A1c, *BMI* Body mass index, *WHtR* Waist-to-height ratio, *BRI* Body roundness index, *ABSI* A body shape index, *WWI* Weight-adjusted waist index, *CI* Conicity index, *WC* Waist circumference

### Association between anthropometric indices and CMM

Table 2 presented the relationship between quartiles of anthropometric indices and CMM risk. All indices were categorized into quartiles, with the lowest quartile (*Q*1) serving as the reference group.

**Table 2:** Association between anthropometric indices and CMM: results from logistic regression analyses

Characteristic	Crude Model		Model Ⅰ		Model Ⅱ	
	OR (95%CI)	*P*-value	OR (95%CI)	*P*-value	OR (95%CI)	*P*-value
BMI category						
* Q*1	Ref		Ref		Ref	
* Q*2	2.35 (1.64, 3.38)	< 0.001	2.37 (1.56, 3.61)	< 0.001	2.55 (1.65, 3.93)	< 0.001
* Q*3	4.27 (2.97, 6.13)	< 0.001	3.20 (2.07, 4.93)	< 0.001	3.04 (1.93, 4.81)	< 0.001
* Q*4	8.75 (5.96, 12.84)	< 0.001	6.55 (4.14, 10.37)	< 0.001	5.89 (3.62, 9.57)	< 0.001
WHtR category						
* Q*1	Ref		Ref		Ref	
* Q*2	2.31 (1.63, 3.29)	< 0.001	2.26 (1.49, 3.42)	< 0.001	2.26 (1.49, 3.42)	< 0.001
* Q*3	3.11 (2.18, 4.43)	< 0.001	2.54 (1.65, 3.91)	< 0.001	2.54 (1.65, 3.91)	< 0.001
* Q*4	5.78 (4.00, 8.34)	< 0.001	4.54 (2.87, 7.16)	< 0.001	4.54 (2.87, 7.16)	< 0.001
BRI category						
* Q*1	Ref		Ref		Ref	
* Q*2	2.31 (1.63, 3.29)	< 0.001	2.26 (1.49, 3.42)	< 0.001	2.26 (1.49, 3.42)	< 0.001
* Q*3	3.11 (2.18, 4.43)	< 0.001	2.54 (1.65, 3.91)	< 0.001	2.54 (1.65, 3.91)	< 0.001
* Q*4	5.78 (4.00, 8.34)	< 0.001	4.54 (2.87, 7.16)	< 0.001	4.54 (2.87, 7.16)	< 0.001
ABSI category						
* Q*1	Ref		Ref		Ref	
* Q*2	1.48 (1.06, 2.07)	0.022	1.57 (1.06, 2.34)	0.025	1.62 (1.07, 2.44)	0.023
* Q*3	1.28 (0.92, 1.79)	0.148	1.16 (0.78, 1.74)	0.459	1.13 (0.74, 1.71)	0.571
* Q*4	1.46 (1.04, 2.04)	0.027	1.20 (0.79, 1.81)	0.396	1.21 (0.78, 1.86)	0.397
WWI category						
* Q*1	Ref		Ref		Ref	
* Q*2	1.74 (1.24, 2.44)	0.001	1.81 (1.21, 2.72)	0.004	1.67 (1.10, 2.54)	0.016
* Q*3	1.82 (1.30, 2.55)	0.020	1.73 (1.14, 2.63)	0.011	1.59 (1.03, 2.45)	0.038
* Q*4	1.88 (1.34, 2.63)	< 0.001	1.51 (0.97, 2.37)	0.071	1.38 (0.86, 2.20)	0.178
CI category						
* Q*1	Ref		Ref		Ref	
* Q*2	1.45 (1.03, 2.03)	0.032	1.58 (1.06, 2.36)	0.024	1.48 (0.98, 2.25)	0.062
* Q*3	1.96 (1.40, 2.76)	< 0.001	1.70 (1.13, 2.56)	0.011	1.59 (1.04, 2.44)	0.032
* Q*4	2.68 (1.90, 3.78)	< 0.001	1.88 (1.24, 2.87)	0.003	1.73 (1.11, 2.70)	0.015
WC category						
* Q*1	Ref		Ref		Ref	
* Q*2	2.12 (1.48, 3.04)	< 0.001	1.83 (1.21, 2.77)	0.004	1.82 (1.19, 2.80)	0.006
* Q*3	3.16 (2.22, 4.51)	< 0.001	2.38 (1.57, 3.63)	< 0.001	2.36 (1.51, 3.68)	< 0.001
* Q*4	9.25 (6.29, 13.63)	< 0.001	5.83 (3.72, 9.14)	< 0.001	5.63 (3.46, 9.15)	< 0.001

The crude model without adjustment; Model I, adjusted for age, sex, marital status, education level, smoking, alcohol consumption, diabetes, hypertension, heart disease, and stroke; and Model II adjusted for age, sex, marital status, education level, smoking, alcohol consumption, diabetes, hypertension, heart disease, stroke, total cholesterol, triglycerides, high-density lipoprotein cholesterol, low-density lipoprotein cholesterol, estimated glomerular filtration rate, serum uric acid and hemoglobin A1c

*CMM* Cardiometabolic multimorbidity, *WHtR* Waist-to-height ratio, *BRI* Body roundness index, *ABSI* A body shape index, *WWI* Weight-adjusted waist index, *CI* Conicity index, *WC* Waist circumference

In the crude model, all anthropometric indices demonstrated positive associations with CMM risk, exhibiting increasing trends from the lowest to highest quartiles Except for ABSI, only *Q*2 and *Q*4 exhibited positive associations with CMM risk. In Model I, the associations remained significant for most indices. Compared to the lowest quartile, BMI showed ORs of 2.37 (1.56, 3.61), 3.20 (2.07, 4.93), and 6.55 (4.14, 10.37) for *Q*2, *Q*3, and *Q*4, respectively. WHtR and BRI demonstrated similar patterns, with the highest quartile showing ORs of 4.54 (2.87, 7.16) for both indices. CI and WC also showed significant associations across quartiles, with *Q*4 having ORs of 1.88 (1.24, 2.87), 5.83 (3.72, 9.14), respectively. In Model II, BMI, WHtR, BRI, CI and WC maintained significant associations across higher quartiles. Compared with the BMI *Q*1 group, the risk of CMM in *Q*2 group increased by 1.55 times (OR = 2.55, 95%CI = 1.65, 3.93, *P* < 0.001); the risk in *Q*3 group increased by 2.04 times (OR = 3.04, 95%CI = 1.93, 4.81, *P* < 0.001); and the risk in *Q*4 group increased by 4.89 times (OR = 5.89, 95%CI = 3.62, 9.57, *P* < 0.001). Compared with the WHR *Q*1 group, the risk of CMM in *Q*2 group increased by 1.26 times (OR = 2.26, 95%CI = 1.49, 3.42, *P* < 0.001); the risk in *Q*3 group increased by 1.54 times (OR = 2.54, 95%CI = 1.65, 3.91, *P* < 0.001); and the risk in *Q*4 group increased by 3.54 times (OR = 4.54, 95%CI = 2.87, 7.16, *P* < 0.001). Similar results were found in BRI. Compared with the CI *Q*1 group, the risk in *Q*3 group increased by 0.59 times (OR = 1.59, 95%CI = 1.04, 2.44, *P* = 0.032); and the risk in *Q*4 group increased by 0.73 times (OR = 1.73, 95%CI = 1.11, 2.70, *P* = 0.015). Compared with the WC *Q*1 group, the risk of CMM in *Q*2 group increased by 0.82 times (OR = 1.82, 95%CI = 1.19, 2.80, *P* = 0.006); the risk in *Q*3 group increased by 1.36 times (OR = 2.36, 95%CI = 1.51, 3.68, *P* < 0.001); and the risk in *Q*4 group increased by 4.63 times (OR = 5.63, 95%CI = 3.46, 9.15, *P* < 0.001). For ABSI, only the *Q*2 group was associated with the risk of CMM, with an OR of 1.62 (95% CI: 1.07, 2.44). For WWI, only the *Q*2 and *Q*3 groups were associated with the risk of CMM, with ORs of 1.67 (95% CI: 1.10, 2.54) and 1.59 (95% CI: 1.03, 2.45), respectively.

### Dose-response relationships between anthropometric indices and CMM

**Fig. 2:**
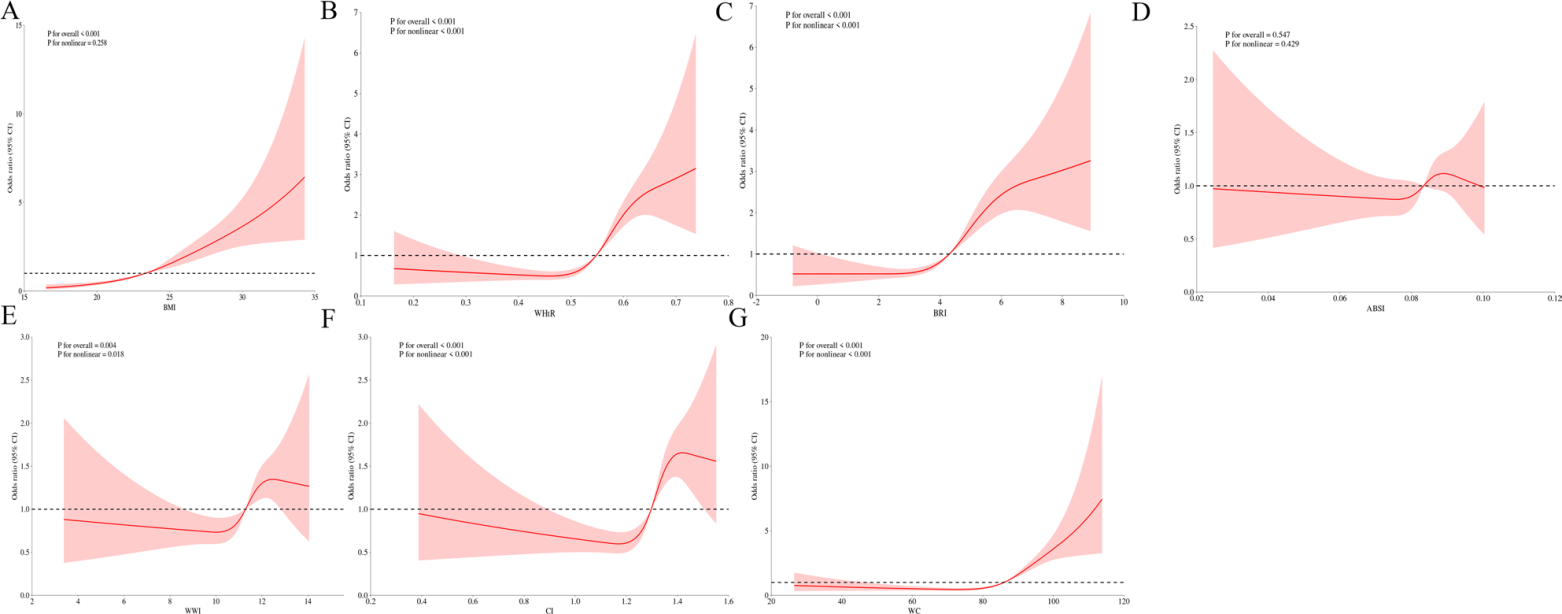
Association between the anthropometric indices and CMM visualized by restricted cubic spline analysis. Adjusted for age, sex, marital status, education level, smoking, alcohol consumption, diabetes, hypertension, heart disease, stroke, total cholesterol, triglycerides, high-density lipoprotein cholesterol, low-density lipoprotein cholesterol, estimated glomerular filtration rate, serum uric acid and hemoglobin A1

Figure 2A-G illustrated the dose-response relationships between anthropometric indices and CMM risk using RCS analysis. Overall, the RCS confirmed that BMI, WHtR, BRI, WWI, CI and WC had significant dose-response relationships with CMM risk. BMI (*P* for overall < 0.001, *P* for nonlinear = 0.258) showed a linear relationship with CMM risk, WHtR (*P* for overall < 0.001, *P* for nonlinear < 0.001), BRI (*P* for overall < 0.001, *P* for nonlinear < 0.001), WWI (*P* for overall = 0.004, *P* for nonlinear = 0.018), CI (*P* for overall < 0.001, *P* for nonlinear < 0.001) and WC (*P* for overall < 0.001, *P* for nonlinear < 0.001) showed a nonlinear relationship with CMM risk. BMI demonstrated a linear relationship across the entire range. WHtR, BRI, WWI, CI and WC all showed a U-shaped association with CMM risk.

### Predictive performance of anthropometric indices for CMM

**Fig. 3:**
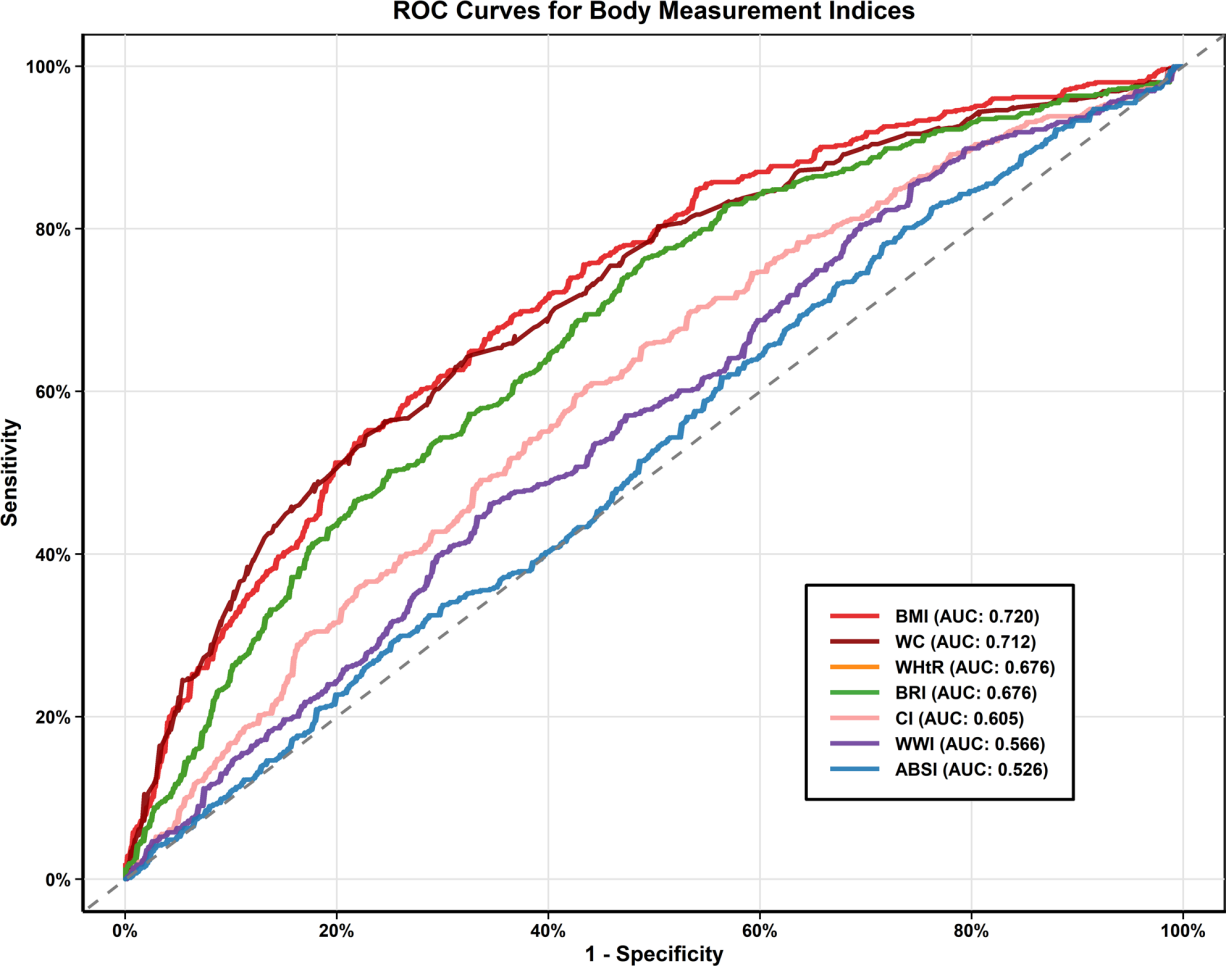
Receiver operating characteristic curves comparing the predictive performance of six anthropometric indices for CM

Table 3; Fig. 3 presented the ROC analysis results evaluating the discriminative performance of six anthropometric indices for predicting CMM.

**Table 3:** The predictive performance of anthropometric indices for CMM

Variable	AUC (95% CI)	Sensitivity	Specificity	Youden Index
BMI	0.720 (0.690–0.749)	0.691	0.635	0.327
WHtR	0.676 (0.644–0.707)	0.764	0.509	0.273
BRI	0.676 (0.644–0.707)	0.764	0.509	0.273
ABSI	0.526 (0.492–0.560)	0.801	0.264	0.065
WWI	0.566 (0.532–0.599)	0.462	0.655	0.117
CI	0.605 (0.572–0.638)	0.605	0.565	0.170
WC	0.712 (0.682–0.742)	0.643	0.677	0.319

*AUC* Area under the curve, *BMI* Body mass index, *WHtR* Waist-to-height ratio, *BRI* Body roundness index, *ABSI* A body shape index, *WWI* Weight-adjusted waist index, *CI* Conicity index, *WC* Waist circumference

The analysis demonstrated that BMI achieved the highest performance with identical AUC values of 0.720 (0.690–0.749), followed by WC with an AUC of 0.712 (0.682–0.742). Both WHtR and BRI had an AUC of 0.676 (0.644–0.707). CI showed an AUC of 0.605 (0.572–0.638), while WWI achieved an AUC of 0.566 (0.532–0.599). ABSI had an AUC of 0.526 (0.492–0.560). ABSI showed the highest sensitivity with a value of 0.801. WC showed the highest specificity, at 0.677. The Youden’s Index values for BMI and WC were 0.327 and 0.319, respectively, indicating balanced sensitivity and specificity. These two indices demonstrated comparable performance in identifying individuals at risk for CMM. The ROC curves showed that BMI and WC consistently outperformed the diagonal reference line, indicating their utility as screening tools for CMM risk assessment. Among all anthropometric indices, BMI and WC had better comprehensive predictive efficacy.

### Predictive performance of anthropometric indices for specific types of CMM

Since CMM consists of two or more diseases of diabetes, heart disease, and stroke, this study further categorized CMM into three subtypes: heart disease + stroke, heart disease + diabetes, and stroke + diabetes. As shown in Table 4, BMI and WC exhibited superior predictive performance whether in predicting those specific types of CMM. Specifically, in the “heart disease + stroke” subgroup, the AUC of BMI was 0.610 (95% CI: 0.570–0.650), while the AUC of WC was 0.603 (95% CI: 0.562–0.643). In the “diabetes + heart disease” subgroup, the AUC of BMI reached 0.690 (95% CI: 0.656–0.724), and the AUC of WC was 0.687 (95% CI: 0.652–0.722). While in the “diabetes + stroke” subgroup, the AUC of BMI was 0.658 (95% CI: 0.613–0.704), and the AUC of WC was even higher at 0.671 (95% CI: 0.625–0.718). This indicates that BMI and WC possess relatively stable and favorable predictive value across different CMM subgroups.

**Table 4:** The predictive performance of anthropometric indices for specific types of CMM

Variable	AUC (95% CI)	Sensitivity	Specificity		Youden Index
Heart disease + stoke					
BMI	0.610 (0.570–0.650)	0.441		0.749	0.190
WHtR	0.573 (0.532–0.614)	0.476		0.659	0.135
BRI	0.573 (0.532–0.614)	0.476		0.659	0.135
ABSI	0.514 (0.471–0.557)	0.458		0.474	-0.068
WWI	0.496 (0.453–0.538)	0.361		0.602	-0.037
CI	0.528 (0.486–0.571)	0.346		0.711	0.057
WC	0.603 (0.562–0.643)	0.567		0.611	0.178
Diabetes + heart disease					
BMI	0.690 (0.656–0.724)	0.726		0.565	0.291
WHtR	0.662 (0.627–0.697)	0.444		0.817	0.261
BRI	0.662 (0.627–0.697)	0.448		0.817	0.265
ABSI	0.542 (0.504–0.580)	0.421		0.657	0.078
WWI	0.575 (0.538–0.612)	0.294		0.840	0.134
CI	0.606 (0.569–0.644)	0.642		0.529	0.171
WC	0.687 (0.652–0.722)	0.748		0.542	0.290
Diabetes + stoke					
BMI	0.658 (0.613–0.704)	0.579		0.687	0.266
WHtR	0.645 (0.597–0.692)	0.495		0.748	0.243
BRI	0.645 (0.597–0.692)	0.494		0.748	0.242
ABSI	0.558 (0.510–0.606)	0.273		0.830	0.103
WWI	0.583 (0.535–0.631)	0.619		0.551	0.170
CI	0.613 (0.566–0.660)	0.519		0.694	0.213
WC	0.671 (0.625–0.718)	0.634		0.680	0.314

*AUC* Area under the curve, *BMI* Body mass index, *WHtR* Waist-to-height ratio, *BRI* Body roundness index, *ABSI* A body shape index, *WWI* Weight-adjusted waist index, *CI* Conicity index, *WC* Waist circumference

## Discussion

This study, utilizing data from middle-aged and older Chinese adults followed for 7 years, provides novel evidence comparing the predictive performance of seven anthropometric indices for CMM. This study demonstrated that BMI and WC exhibited superior discriminative ability compared to novel indices, with two maintaining significant dose-response relationships with CMM risk after comprehensive adjustment for confounders. Furthermore, BMI and WC also showed remarkable efficacy in predicting specific CMM subtypes. These results further highlighted the key preventive role of weight and WC management in addressing CMM and its subtypes, providing evidence for formulating health promotion strategies.

The 7.42% incidence of CMM observed in our cohort over the 7-year follow-up timeframe underscores the substantial burden of CMM in middle-aged and older Chinese adults. This finding aligns with recent reports from China showing increasing prevalence of multimorbidity, particularly cardiometabolic conditions, in aging populations [23, 24]. Given the heavy burden of CMM, identifying effective predictive indicators is crucial for early screening and intervention. Notably, multiple studies have begun to explore and compare the predictive efficacy of different anthropometric indices for CMM. Lu et al. reported in a national longitudinal cohort study of 10,521 Chinese adults that WHtR, WC, and waist divided by height^0.5 showed better abilities in predicting CMM than BMI among middle-aged and older Chinese adults [25]. Similarly, Qin et al. found in a cross-sectional study of 101,973 Chinese adults that WHtR and BRI had higher discriminative values for CMM (AUCs: 0.679 for both) compared to BMI [26]. The systematic review and meta-analysis by Ashwell et al. demonstrated that WHtR was a better screening tool than WC and BMI for adult cardiometabolic risk factors, with WHtR improving discrimination by 4–5% over BMI (*P* < 0.01) [27]. However, our study results exhibited partial discrepancies from these previous studies, as we observed that BMI and WC outperformed other indices in predicting CMM risk, both for overall CMM and its specific types. This discrepancy may stem from two factors. First, there are differences in the adjustment of confounders across studies: our study employed PSM to balance baseline characteristics and further adjusted for multiple metabolic confounders in the model. This approach may have reduced residual confounding, thereby clarifying the true predictive value of each index. Second, differences in population stratification may also contribute to this inconsistency: although all studies focused on Chinese adults, our sample had a more homogeneous age distribution among the “middle-aged and older” population (after PSM), whereas previous cohorts may have included a broader age range (encompassing younger middle-aged adults). This difference could alter the relative predictive ability of each index.

The core pathogenesis of CMM lies in excessive systemic fat accumulation (which induces insulin resistance and chronic inflammation) and abdominal visceral fat accumulation (which releases free fatty acids and pro-inflammatory factors to directly damage metabolic organs) [28], while BMI and WC precisely correspond to these two pathological key points: specifically, BMI directly quantifies systemic adiposity (total fat mass) via the formula “weight/height²”, and its linear dose-response relationship with CMM confirms that it fully aligns with th logic of “the greater the total fat mass, the heavier the metabolic burden” [29]; at the same time, WC directly measures abdominal circumference to accurately reflect visceral fat content (over 70% of abdominal fat is visceral fat), and its U-shaped dose-response relationship (risk rises accelerated after exceeding the threshold) exactly corresponds to the pathological process where metabolic disorders worsen sharply once visceral fat exceeds the physiological compensation limit [30]. The superior predictive performance of BMI and WC is more in line with the practical needs of CMM prevention and control in middle-aged and older Chinese populations—there is no need to pursue novel indices with complex formulas, but instead to choose classic indices that accurately reflect pathology, are simple, and easy to popularize, which is precisely the core of their clinical value.

In our study, WHtR and BRI demonstrated comparable predictive capacity for CMM. This finding has been consistently replicated in numerous independent investigations. A study conducted in a Chinese cohort demonstrated consistent predictive performance of WHtR and BRI for hypertension, dyslipidemia, type 2 diabetes, and multimorbidity [31]. A parallel finding was observed in a study focused on predicting metabolic syndrome among middle-aged and older Chinese adults [32]. This situation may likely attributable to their shared sensitivity in quantifying abdominal fat accumulation—particularly visceral adiposity [33, 34], and visceral fat is precisely the core driver of cardiometabolic pathology. BRI utilizes a more complex computational algorithm to estimate visceral adiposity, its predictive performance did not surpass that of WHtR, implying that simpler indices may offer greater practical advantage in clinical settings.

Our finding that ABSI showed poor discriminative ability aligns with observations from other populations. Zhang et al. in their large cross-sectional study of 101,973 Chinese adults also found that ABSI had the weakest discriminative power for CMM compared to other indices [26]. A Polish population-based study similarly found that ABSI demonstrated the weakest predictive capacity for metabolic syndrome across both male and female cohorts [35]. ABSI was originally derived from anthropometric characteristics of the US population. Variations in body morphology and adipose distribution patterns across ethnic groups result in its suboptimal applicability to both Asian and European populations. This suggests that although the ABSI was originally designed to reflect abnormal body fat distribution by combining WC, BMI, and height, its actual efficacy may have significant limitations across different ethnic populations. Moreover, compared with WHtR, ABSI is less sensitive in quantifying visceral fat, and its algorithm may fail to fully capture the fat distribution characteristics most directly related to metabolic risk.

Although this study is the first to explore the predictive efficacy of seven anthropometric indicators for CMM in a Chinese population, several limitations exist. First, the definition of CMM relies on self-reported physician diagnoses, which may introduce various biases. Specifically, an overly broad case definition (that may include non-metabolism-related heart conditions) can dilute the association between anthropometric measures and CMM, whereas misreporting or underreporting may lead to risk estimates biased toward the null, thereby underestimating the true strength of the association [36]. Second, the study sample was limited to Chinese adults, so the extent to which the results can be generalized to other ethnic populations may be restricted—though recent studies have shown that the WHtR has universal applicability across different ethnic groups. Third, the study lacked medication data, and medication use may affect the association between anthropometric measures and cardiometabolic diseases. Fourth, this study used logistic regression rather than the Cox proportional hazards model. The former cannot utilize information on event timing and only focuses on whether an event occurred at the end of follow-up, which may result in a loss of statistical power [37]. Furthermore, when the cumulative incidence of events is high (> 10%), OR may overestimate relative risks [38, 39]. Finally, the assessment of baseline anthropometric measures did not account for their temporal changes, and such changes may influence the occurrence and progression of CMM.

This large retrospective study demonstrated that BMI and WC were superior to novel anthropometric indices for predicting CMM risk among Chinese middle-aged and older adults. This finding further supports the value of BMI and WC in identifying individuals at high risk of CMM and reinforces their role as practical tools for this specific condition.

## Data Availability

The datasets analyzed during the current study are available in the CHARLS and can be accessed at the following website: https://charls.pku.edu.cn/.
